# Loss of microstructural integrity in left hemispheric white matter tracts is associated with poorer digits in noise understanding

**DOI:** 10.1007/s11357-025-01707-5

**Published:** 2025-05-26

**Authors:** Jordi H. C. Boons, Gertjan Dingemanse, Elisabeth J. Vinke, Bernd Kremer, Meike W. Vernooij, André Goedegebure

**Affiliations:** 1 Department of Otorhinolaryngology, Head and Neck Surgery, Erasmus , P.O. Box 2040, MC 3000 CA Rotterdam, The Netherlands; 2 Department of Radiology and Nuclear Medicine, Erasmus MC, Rotterdam, The Netherlands; 3 Department of Epidemiology, Erasmus MC, Rotterdam, The Netherlands

**Keywords:** Age-related hearing loss, Digits-in-noise, DTI, Population-based, White matter tracts

## Abstract

Age-related hearing loss (ARHL) is a prevalent condition among older adults and is regarded as a potentially modifiable risk factor for dementia. Although multiple studies have investigated pure-tone thresholds as a measure for ARHL and its relationship to dementia, the potential role of the central auditory system has received little attention. To address this gap in the literature, this study investigates the relationship between central auditory functioning, assessed using the speech-reception-threshold (SRT) of the digits-in-noise (DIN) test, and the microstructural integrity of white matter tracts in the Rotterdam Study. A total of 1669 participants underwent the DIN test and had diffusion imaging data available. The SRT was found to be significantly associated with the microstructural integrity of the left inferior fronto-occipital fasciculus, inferior longitudinal fasciculus, and posterior thalamic radiation. After accounting for audibility effects, the association with the inferior fronto-occipital fasciculus and the inferior longitudinal fasciculus was even stronger, while the association with the posterior thalamic radiation was no longer significant. These findings suggest that age-related declines in specific brain regions may contribute to difficulties in speech-in-noise understanding among the elderly.

**Supplementary Information:**

The online version contains supplementary material available at 10.1007/s11357-025-01707-5.

## Introduction

Age-related hearing loss (ARHL) is a highly prevalent condition among the aging population and has recently been recognized as a potentially modifiable risk factor for dementia [1]. Several studies have examined elevated pure-tone thresholds as a measure of ARHL and its association with dementia, as well as brain structures as a measure of brain health [2–5]. However, the role of the central auditory system on brain health remains underexplored. The central auditory system processes auditory signals received by the ears and extracts relevant information from these signals. Aging may affect central auditory processes such as auditory discrimination, binaural processing, and temporal processing [6]. It is particularly relevant to study central aspects of hearing in relation to brain health, as dysfunction of the central auditory system may be associated with an increased risk of cognitive decline and dementia [7].

The central auditory system includes temporal, parietal, and frontal brain regions [8]. Communication between these regions is facilitated by white matter (WM) pathways. These can be divided into a dorsal stream and a ventral stream [9]. The dorsal stream is responsible for sounds-to-motor mapping, while the ventral stream, consisting of the inferior fronto-occipital fasciculus and uncinate fasciculus, decodes sounds into a meaningful message [9, 10]. These functions may be impaired when the WM microstructure is affected [11]. With diffusion-weighted imaging (DWI), diffusion of water molecules along the tracts can be measured. From these images, diffusion tensors can be estimated to obtain and quantify microstructural properties and organization of WM tracts with measures such as fractional anisotropy (FA) and mean diffusivity (MD) [11]. FA reflects the degree of anisotropy; it quantifies the directional movement along the axonal fibers and may reflect effective myelination and axonal integrity [12]. MD represents the mean diffusion of each direction and is associated with myelin loss and axonal damage [13].

Speech-in-noise tests are useful for assessing aspects of central auditory function because they reflect not only hearing thresholds (i.e., the audibility of speech), but also the ability to process speech sounds when it is partially masked by noise. These tests may therefore reveal additional associations with WM microstructure compared to pure-tone thresholds alone. In addition to hearing thresholds and central auditory function, speech-in-noise recognition is influenced by cognitive abilities such as working memory, attention, and inhibition [14]. When investigating the relationship between central auditory function and brain health, cognition may act as a confounder as it is related to both terms [15, 16]. The digits-in-noise (DIN) test, whose outcome is minimally influenced by cognition, may limit confounding by cognitive abilities when investigating the relationship between central auditory processing and brain health [17, 18].

In light of the hypothesized relationship between poorer central auditory processing and cognitive decline [7], it is of great interest to investigate the relationship between speech understanding and microstructural integrity of WM tracts through the use of diffusion tensor imaging (DTI). Two studies have examined these associations in aging populations, of which both identified an association between speech-in-noise understanding and WM microstructure in the uncinate fasciculus [3, 19]. While these results appear promising, it is unclear to what extent their findings reflect central auditory processing instead of peripheral hearing loss. Both studies used a relatively simple linear correction model based on average pure-tone thresholds to account for reduced audibility. As age-related hearing loss generally has a sloping audiogram, correction with average thresholds may lead to an inaccurate estimation of speech audibility across the entire frequency range. Therefore, it is likely that their findings reflect effects of peripheral hearing on WM microstructure. Moreover, Armstrong et al. used the QuickSIN test, the outcome of which is related to cognitive performance [20], introducing a potential influence of cognition.

The aim of our study is to investigate the relationship between speech understanding in noise and microstructural integrity of WM-tracts, focusing on central auditory processing instead of aspects related to peripheral hearing, such as audibility, or general cognition. To achieve this, audibility effects were corrected with a comprehensive validated correction model, the speech intelligibility index, and the cognitive undemanding DIN test was used. We expect to find the strongest associations between speech-in-noise understanding and WM microstructure in the uncinate fasciculus and inferior fronto-occipital fasciculus, which constitute the ventral auditory stream, given their role in decoding sounds into a meaningful message [9, 10]. By carefully addressing influences of both peripheral hearing and cognition, this study seeks to further clarify how central auditory processing relates to WM microstructure, potentially providing new insights into the links between central auditory function and brain health.

## Methods

### Study population

This study is part of the Rotterdam Study, a large population-based prospective cohort study. The aim of the Rotterdam Study is to investigate the determinants and consequences of aging. Further details are described elsewhere [21]. The study population consists of residents aged 45 years and older from the Ommoord area, a suburb of Rotterdam, the Netherlands. All participants were examined at the research center at study enrollment and subsequent visits every 3–6 years. Since 2005, brain MRI has been included in the core protocol of the Rotterdam Study. Hearing assessment with pure-tone audiometry and the DIN test has been included in the core protocol of the Rotterdam Study since the end of 2011.

The population included in this study consists of 2329 participants from cohorts RS-II and RS-III who underwent hearing assessment and brain MRI scanning between 2011 and 2014. As we are only interested in central auditory aspects apart from effects from reduced audibility, we excluded participants with hearing loss for whom the SRT mainly refers to hearing thresholds (pure-tone average (PTA) > 25 dB HL, see section “Digits in noise test”) (*N* = 632). In addition, participants with conductive hearing loss in the better hearing ear (air–bone gap ≥ 15 dB; *N* = 2) and with a vestibular schwannoma (*N* = 4) were excluded, leaving 1691 participants. The median time between audiometric assessment and the brain MRI scan was 0.15 years (IQR [0.10–0.23]).

### Pure-tone audiometry

Pure-tone audiometry was performed in a soundproof booth to determine hearing loss by measuring hearing thresholds in decibel hearing level (dB HL) [21]. A computer-based audiometry system (Decos Technology Group, version 210.2.6, AudioNigma interface) and TDH-39 headphones were used. Calibration was performed annually according to ISO-standard 389 [22]. Hearing levels were measured according to ISO-standard 8253–1 [23]. Air conduction thresholds (0.25–8 kHz) and bone conduction thresholds (0.5 and 4 kHz) were measured for both ears, with masking according to the method of Hood if necessary [24]. The best hearing ear was determined by averaging the thresholds across all frequencies and identifying the ear with the lowest average hearing threshold of both ears. In the case of equal averages, the left or right ear was chosen alternately. The PTA of the better ear was calculated for the frequencies 0.5, 1, 2, and 4 kHz.

### Digits-in-noise test

We used the DIN test to measure speech understanding in noise by presenting 24 pre-recorded male-spoken digit triplets to the best hearing ear at varying levels of speech-to-noise ratio (SNR). The noise was kept constant at 55 dB SPL throughout the test, while the speech level was varied to achieve a given SNR. We presented the triplets using the same hardware as for the pure-tone audiometry with annually calibrated sound levels for both speech and noise. First, the initial speech level was determined by repeatedly presenting the first triplet, increasing the level by 4 dB, until it was heard correctly. The measurement then followed an adaptive up-down procedure, using 2-dB steps. A correct response results in a 2-dB decrease in SNR for the next triplet, while an incorrect response results in a 2-dB increase in SNR for the next digit triplet. This procedure was repeated for all 24 digit triplets. We calculated the SRT as the mean of the SNRs of the last 20-digit triplets. To assess the accuracy of the SRT measurements, we determined the measurement error by taking the standard deviation of all individual within-test SNRs. Participants whose within-test standard deviation exceeded 3.7 dB (3 standard deviations above the mean) were identified as outliers and excluded from further analysis (*N* = 22).

Normal-hearing listeners achieve a 100% speech recognition score of digit triplets in quiet at 30 dB(A) (~ 33 dB SPL) [17]. At the 55-dB SPL presentation level of the DIN test, it is possible that participants with elevated hearing thresholds may not achieve a 100% score in quiet, which could affect the SRT measured in noise. Therefore, we excluded participants with a PTA greater than 25 dB HL (~ 55–33 dB).

### Speech intelligibility index

However, even with a PTA below 25-dB HL, it is still possible that audibility was not optimal in a smaller frequency range, such as the high frequencies (4–8 kHz). To correct for these effects of increased hearing thresholds on the SRT, we applied a commonly used model, the speech intelligibility index (SII). The SII is a measure of the proportion of speech information in a speech-in-noise signal that is usable by the listener, expressed as a value between 0 and 1 [25]. The formula is defined as $SII= \sum_{i=1}^{n=18}{A}_{i}{I}_{i}$, where *i* is the frequency band, *I* is importance, and *A* is audibility. Audibility is a measure, between 0 and 1, of the proportion of the speech dynamic range that contributes to speech intelligibility within a band [25]. It is determined by the speech level, the noise level, and the hearing threshold in a band. The one-third octave frequency band method was used. We measured the 1/3 octave band levels of the speech signal and the noise with a 6 cc coupler and used a transfer function to convert these values to free-field levels (Bentler et al. 1989). We used the standard band importance coefficients.

### MRI acquisition and processing

Multi-sequence MRI was performed on a single 1.5-Tesla MRI scanner (GE Signa Excite, Milwaukee, USA) with a dedicated 8-channel head coil with the possibility for parallel imaging, keeping hardware and software setup unchanged over the entire study period. Further details on the scan protocol and sequence details are described elsewhere [26]. In summary, the protocol included a T1-weighted image (acquisition time 6:24 min, repetition time (TR) 13.8 ms, echo time (TE) 2.8 ms, voxel size 0.49 × 0.49 × 1.6 mm^3^), a T2-weighted fluid-attenuated inversion recovery (FLAIR) sequence image (acquisition time 6:25 min, TR 8000 ms, TE 120 ms, voxel size 0.78 × 1.12 × 1.6 mm^3^), a proton density-weighted image (acquisition time 6:09 min, TR 12,300 ms, TE 17.3 ms, voxel size 0.6 × 0.98 × 1.6 mm^3^), and a single-shot diffusion-weighted image (acquisition time 3:44 min, TR 8000 ms, TE 74.6 ms, voxel size 3.3 × 2.2 × 3.5 mm^3^, field-of-view 21 cm2, matrix 96 × 64 (interpolated to 256 × 256), slice thickness 3.5 mm, 36 contiguous slices, applying parallel imaging (array spatial sensitivity encoding technique) with acceleration factor = 2). For the diffusion image, gradients of *b* = 1000 s/mm^2^ were applied in 25 non-collinear directions, and three volumes were acquired without diffusion weighting (*b* = 0 s/mm^2^).

A number of preprocessing steps were performed before analysis. To summarize, structural scans for each participant were spatially coregistered using rigid registration. Brain masking and nonuniformity corrections were applied to the scans, after which they were segmented in gray matter, white matter, cerebrospinal fluid, and background tissue using an approach based on k-nearest neighbor segmentation approach on the T1-weighted and proton density images [27]. Intracranial volume (ICV) was defined as the sum of white and gray matter and cerebrospinal fluid volumes, excluding the cerebellum and surrounding cerebrospinal fluid. White matter hyperintensities (WMHs) were segmented with an in-house developed automated segmentation method using a 2-step protocol which relies on the brain tissue segmentation and the FLAIR image [28].

**Fig. 1:**
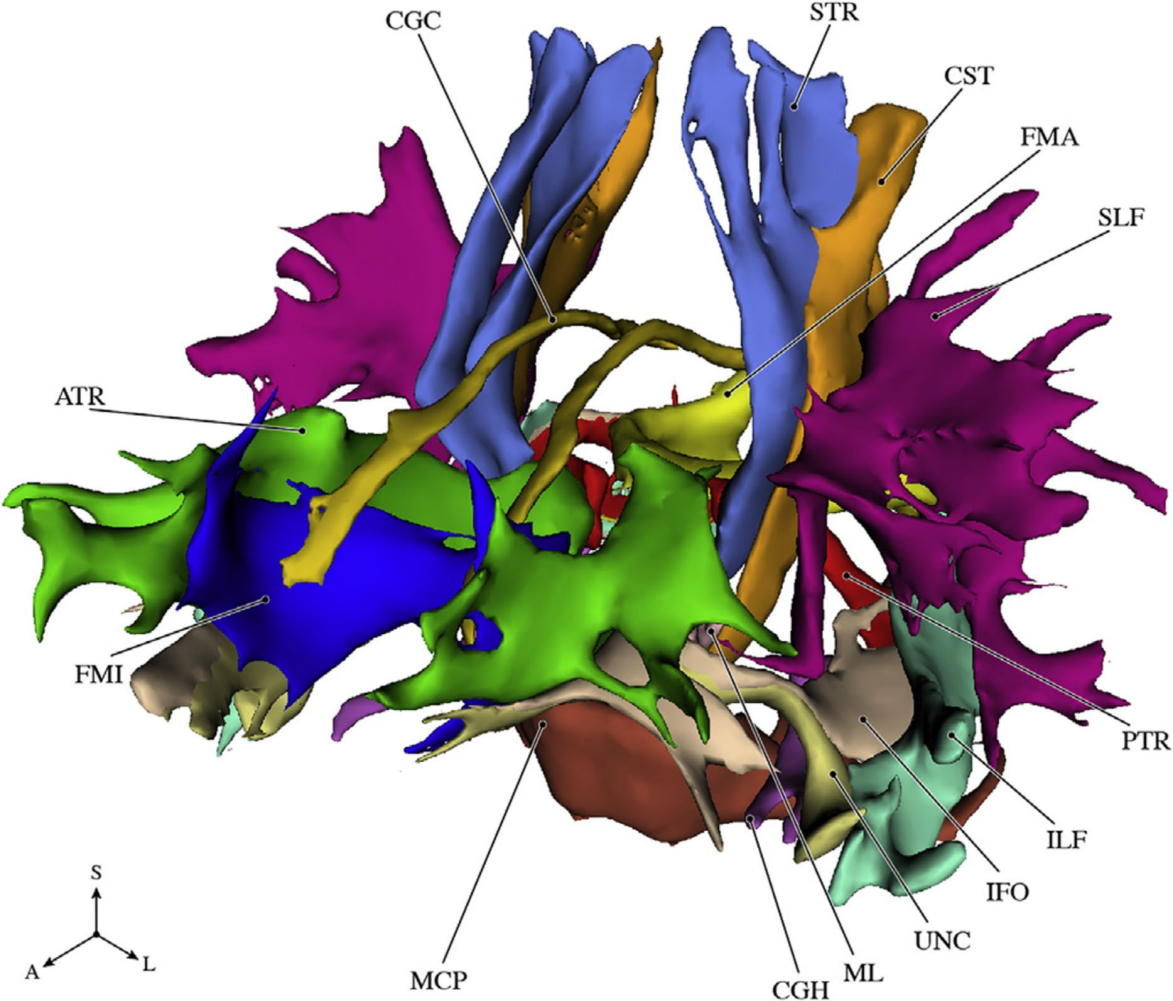
Schematic overview of the 14 white matter tracts. Abbreviations: A, anterior; ATR, anterior thalamic radiation; CGC, cingulate gyrus part of cingulum; CGH, parahippocampal part of cingulum; CST, corticospinal tract; FMA, forceps major; FMI, forceps minor; IFO, inferior fronto-occipital fasciculus; ILF, inferior longitudinal fasciculus; L, lateral; MCP, middle cerebellar peduncle; ML, medial lemniscus; PTR, posterior thalamic radiation; S, superior. Adapted from [34]

All diffusion data were processed using a standardized pipeline [29]. In short, diffusion data were corrected for motion and Eddy currents by affine coregistration with Elastix [30]. Diffusion tensors were estimated using a nonlinear Levenberg–Marquardt estimator in ExploreDTI [31]. FA and MD were computed from the estimated tensor images. Probabilistic tractography was used to segment WM tracts using PROBTRACKX [32]. For each tract, seed, target, stop, and exclusion masks are defined to perform the tractography [33]. Using this approach, we extracted median FA and MD in the normal-appearing WM for 14 different tracts (11 of which were segmented bilaterally) as illustrated in Fig. 1. For the bilateral tracts, FA and MD were computed for the left and right tract separately. Due to tractography failures or (visually) rejected segmentations, tract-specific measurements were missing for on average 6.2 participants (ranging from 2 to 13 participants) per tract. Tract-specific FA and MD were standardized and centered using *z*-scores. We obtained tract-specific WM volumes and tract-specific WMH volumes (natural log-transformed) by combining tissue and tract segmentations [34].

### Other covariates

Information on various covariates was collected at enrollment and reassessed at each follow-up visit [21]. Smoking behavior, educational level, and the Mini-Mental State Examination (MMSE) were assessed in home interviews [35]. Smoking behavior was categorized as never, former, and current smoking. Educational level was defined as completed primary education, lower education, intermediate vocational education, or higher education. Alcohol consumption was self-reported using a comprehensive, validated food-frequency questionnaire, and we calculated the daily alcohol consumption in grams [36]. Systolic and diastolic blood pressure was measured twice on the right arm with a random-zero sphygmomanometer, and the mean of these measurements was used for the analysis. The use of antihypertensive medication was assessed during the home interview. Fasting blood samples were collected for glucose determination using the hexokinase method. The presence of diabetes mellitus was defined as a fasting glucose level greater than 7 mmol/L or a non-fasting glucose level of 11 mmol/L or greater.

### Statistics

Associations between tract-specific DTI markers (FA and MD) and the outcome of the DIN test were investigated with multivariable linear regression models. For the outcome of the DIN test, we use two different measures, the SRT and the SII. The SRT is our main variable of interest, as it is the most commonly used. Results with the SII are expected to be less dependent on hearing thresholds. Therefore, the SII is likely to reflect central auditory hearing better than the SRT. Tract-specific FA and MD were considered the independent variables, while the outcome of the DIN test (i.e., SRT or SII) was the dependent variable. Separate analyses were run with the SII and SRT. Standardized betas and 95% confidence intervals (CIs) were estimated for each tract-specific FA and MD.

In model I, we adjusted for sex, age, age^2^, tract-specific WM volume, natural log-transformed tract-specific WMH volume, ICV, PTA, and time between the hearing assessment and brain MRI acquisition. Model II was extended with the following covariates: educational level, smoking behavior, alcohol consumption, systolic blood pressure, diastolic blood pressure, the use of antihypertensive drugs, and the presence of diabetes mellitus. We adjust for these cardiovascular risk factors based on evidence that impaired cardiovascular health has a negative influence on the central auditory system [31]. Furthermore, previous research specifically described a relationship between cardiovascular risk factors and speech-in-noise understanding [32]. In Model III, we additionally accounted for general cognition with the MMSE to analyze whether the found effects are driven by cognition.

The Lancet report has proposed that the effects of hearing loss on dementia are particularly relevant during midlife (< 65 years of age) [1]. Therefore, we conducted a sensitivity analysis in which we stratified the population with the threshold of 65 years, resulting in a midlife and late-life group. To reduce complexity and limit information overload for the reader, this analysis was restricted to models using the SRT as the dependent variable. In a second sensitivity analysis, we used tract-specific axial diffusivity (AD) and radial diffusivity (RD) as independent variables, while using the same method as for the main analysis with FA and MD. We conducted this sensitivity analysis to investigate whether associations with FA and MD were driven by diffusion changes along the fiber direction (AD) or perpendicular to the fiber (RD).

The *p* value (significance level *α* = 0.05) for multiple testing was determined using an approach in which the number of independent tests is based on the variance of the eigenvalues of the correlation matrix of all variables (tract-specific FA and MD for each tract, *N* = 32). The number of independents tests (*M*_eff_) was calculated using the formula: $M_{eff}=\frac{\left[\sum_{m=1}^m\sqrt{\lambda m}\right]^2}{\sum_{m=1}^m\lambda m}$, in which *M* is the number of variables, ${\lambda }_{\mathrm{m}}$ is the eigenvalues of the correlation matrix. This resulted in *M*_eff_ = 16.15. Applying the Sidák correction for multiple testing ($\propto Sid\overset'{ak=}$)^1/Meff^)), the adjusted significance threshold was set at *p* < 0.0032 [37].

We only included association and projection tracts in the calculation of the significance threshold, because the tracts related to auditory processing are categorized as association tracts. Furthermore, a study investigating the relationship between cognition and FA/MD found significant effects only in association and projection tracts [34]. Thus, any potential cognitive influences on speech perception in our study are likely captured within these tracts.

We included the tracts in the other categories (brainstem, limbic system and callosal tracts) as a verification in the analysis. All statistical analyses have been performed in *R* [38].

## Results

The demographic characteristics of the 1669 participants are shown in Table 1. The median age was 63.1 years (IQR [58.1–67.2]), ranging from 51.7 to 91.8 years. In total, 55.6% of the population were female. The mean MMSE score was 28.4, and 96.7% of the population had a MMSE above 25.

**Table 1:** Demographic characteristics of the study population (*N* = 1669)

Age, years [IQR]	63.2 [58.2–67.2]
Age (range)	51.7–91.8
Female (*N* (%))	945 (56.6%)
Educational level (*N* (%)) Primary Lower Intermediate vocational Higher	99 (5.9%) 571 (34.2%) 487 (29.2%) 501 (30.0%)
Daily alcohol consumption, gram [IQR]	6.4 [0.54–8.6]
Smoking (*N* (%)) Never Former Current	582 (34.9%) 812 (48.7%) 274 (16.4%)
Systolic blood pressure, mmHg [IQR]	135 [123–149]
Diastolic blood pressure, mmHg [IQR]	82 [75–90]
Use of blood pressure lowering medication (*N* (%))	596 (35.7%)
Diabetes mellitus (*N* (%))	120 (7.2%)
MMSE, (SD)	28.4 (1.6)
Pure-tone average, dB HL [IQR]	15.0 [11.2–20.0]
Speech recognition threshold, dB SNR [IQR]	− 6.0 [− 7.0 to − 4.9]
Speech intelligibility index, [IQR]	0.24 [0.22–0.28]
Time between audiometry and MRI scanning, years [IQR]	0.15 [0.10–0.23]

Abbreviation:* dB HL*, decibels hearing level; *IQR*, inter quartile range; *mmHg*, millimeters of mercury; *MRI*, magnetic resonance imaging; *N*, number of participants; *SD*, standard deviation; *SNR*, speech-to-noise ratio

Standardized effect estimates for FA and MD in relationship to the SRT are given in Tables 2 and 3, respectively. Associations were found exclusively in projection and association tracts within the left hemisphere. The effect estimates for model II were either slightly attenuated or essentially unchanged compared to model I. In particular, the effect estimates for the MD of the inferior fronto-occipital fasciculus increased slightly in model II. After controlling for general cognition with the MMSE in Model III, the association with the posterior thalamic radiation was not significant anymore.

**Table 2:** Associations between the SRT of the DIN test and the tract-specific FAs

WM tracts	Model I		Model II		Model III	
	Left hemisphere	Right hemisphere	Left hemisphere	Right hemisphere	Left hemisphere	Right hemisphere
*Brainstem tracts*						
Middle cerebellar peduncle	− 0.029 (− 0.073, 0.015)		− 0.028 (− 0.074, 0.018)		− 0.017 (− 0.063, 0.029)	
Medial lemniscus	0.0094 (− 0.039, 0.058)	0.0067 (− 0.042, 0.055)	0.024 (− 0.025, 0.073)	0.023 (− 0.026, 0.072)	0.026 (− 0.023, 0.075)	0.024 (− 0.025, 0.073)
*Projection tracts*						
Corticospinal tract	0.028 (− 0.018, 0.074)	− 0.0024 (− 0.047, 0.043)	0.04 (− 0.0064, 0.086)	0.0089 (− 0.037, 0.054)	0.039 (− 0.007, 0.085)	0.0092 (− 0.036, 0.054)
Anterior thalamic radiation	− 0.026 (− 0.08, 0.028)	− 0.026 (− 0.082, 0.030)	− 0.025 (− 0.08, 0.030)	− 0.025 (− 0.081, 0.031)	− 0.025 (− 0.080, 0.030)	− 0.025 (− 0.081, 0.031)
Superior thalamic radiation	− 0.011 (− 0.06, 0.038)	− 0.0069 (− 0.054, 0.041)	− 0.0076 (− 0.057, 0.041)	− 0.00029 (− 0.048, 0.047)	− 0.011 (− 0.06, 0.038)	− 0.0002 (− 0.048, 0.047)
Posterior thalamic radiation	− 0.086 *** (− 0.14, − 0.035)	− 0.039 (− 0.092, 0.014)	− 0.079 ** (− 0.13, − 0.028)	− 0.03 (− 0.083, 0.023)	− 0.073 ** (− 0.12, − 0.022)	− 0.035 (− 0.088, 0.018)
*Association tracts*						
Superior longitudinal fasciculus	− 0.046 (− 0.096, 0.004)	0.00018 (− 0.051, 0.051)	− 0.046 (− 0.097, 0.005)	0.0054 (− 0.047, 0.057)	− 0.047 (− 0.097, 0.004)	0.00061 (− 0.051, 0.052)
Inferior longitudinal fasciculus	− 0.098 *** (− 0.15, − 0.049)	− 0.035 (− 0.084, 0.014)	− 0.090 *** (− 0.14, − 0.040)	− 0.029 (− 0.079, 0.021)	− 0.087 *** (− 0.14, − 0.037)	− 0.029 (− 0.078, 0.02)
Inferior fronto-occipital fasciculus	− 0.075 ** (− 0.13, − 0.025)	− 0.05 (− 0.1, − 0.00022)	− 0.075 ** (− 0.13, − 0.024)	− 0.05 (− 0.1, 0.00099)	− 0.073 ** (− 0.12, − 0.022)	− 0.051* (− 0.1, − 0.00028)
Uncinate fasciculus	− 0.052 (− 0.1, 0.00086)	− 0.01 (− 0.06, 0.040)	− 0.053 (− 0.11, 0.00042)	− 0.011 (− 0.061, 0.039)	− 0.05 (− 0.1, 0.0031)	− 0.014 (− 0.064, 0.036)
*Limbic system tracts*						
Cingulate gyrus part of the cingulum	0.018 (− 0.028, 0.064)	− 0.039 (− 0.084, 0.006)	0.015 (− 0.032, 0.062)	− 0.034 (− 0.079, 0.011)	0.01 (− 0.037, 0.057)	− 0.037 (− 0.082, 0.008)
Parahippocampal part of the cingulum	0.0089 (− 0.037, 0.055)	0.011 (− 0.036, 0.058)	0.016 (− 0.03, 0.062)	0.0079 (− 0.039, 0.055)	0.017 (− 0.029, 0.063)	0.0079 (− 0.039, 0.055)
*Callosal tracts*						
Forceps major	− 0.043 (− 0.097, 0.011)		− 0.037 (− 0.092, 0.018)		− 0.038 (− 0.092, 0.016)	
Forceps minor	− 0.012 (− 0.065, 0.041)		− 0.0085 (− 0.062, 0.045)		− 0.0078 (− 0.061, 0.045)	

Values represent the mean differences in *z*-score (95% confidence interval) of the SRT per standard deviation increase of the tract-specific FA. Stars indicate the significance level: * (*p* < 0.05), ** (*p* < 0.01), *** (*p* < 0.001). Results in bold were statistically significant after correction for multiple testing (*p* < 0.0032). Model I: adjusted for sex, age, age^2^, tract-specific WM volume, natural-log-transformed tract-specific WMH volume, ICV, and time between the hearing assessment and brain MRI acquisition. Model II: Model I and additionally adjusted for educational level, smoking behavior, alcohol consumption, systolic blood pressure, diastolic blood pressure, the use of anti-hypertensive drugs and the presence of diabetes mellitus.*DIN*, digits in noise; *FA*, fractional anisotropy; *ICV*, intracranial volume; *MRI*, magnetic resonance imaging; *SRT*, speech reception threshold; *WM*, white matter; *WMH*, white matter hyperintensity

**Table 3:** Associations between the SRT of the DIN test and the tract-specific M Ds

WM tracts	Model I		Model II		Model III	
	Left hemisphere	Right hemisphere	Left hemisphere	Right hemisphere	Left Hemisphere	Right hemisphere
*Brainstem tracts*						
Middle cerebellar peduncle	0.008 (− 0.034, 0.050)		0.0014 (− 0.042, 0.044)		− 0.0011 (− 0.044, 0.042)	
Medial lemniscus	− 0.032 (− 0.075, 0.011)	− 0.015 (− 0.059, 0.029)	− 0.042 (− 0.086, 0.0017)	− 0.027 (− 0.071, 0.017)	− 0.038 (− 0.082, 0.0055)	− 0.022 (− 0.066, 0.022)
*Projection tracts*						
Corticospinal tract	− 0.0002 (− 0.055, 0.055)	0.070 (0.016, 0.12)	− 0.0078 (− 0.064, 0.049)	0.061 (0.0062, 0.12)	− 0.0025 (− 0.059, 0.054)	0.063 * (0.0084, 0.12)
Anterior thalamic radiation	0.048 (− 0.022, 0.12)	0.029 (− 0.04, 0.098)	0.039 (− 0.032, 0.11)	0.024 (− 0.045, 0.093)	0.038 (− 0.033, 0.11)	0.02 (− 0.049, 0.089)
Superior thalamic radiation	0.070 * (0.011, 0.13)	0.052 (− 0.0049, 0.11)	0.068 * (0.0075, 0.13)	0.047 (− 0.011, 0.10)	0.071 * (0.011, 0.13)	0.044 (− 0.013, 0.1)
Posterior thalamic radiation	0.059 * (0.0078, 0.11)	0.036 (− 0.017, 0.089)	0.062 * (0.011, 0.11)	0.033 (− 0.020, 0.086)	0.061 * (0.01, 0.11)	0.029 (− 0.023, 0.081)
*Association tracts*						
Superior longitudinal fasciculus	0.047 (− 0.0071, 0.10)	0.0094 (− 0.045, 0.064)	0.046 (− 0.009, 0.10)	0.0085 (− 0.046, 0.063)	0.047 (− 0.0076, 0.1)	0.011 (− 0.043, 0.065)
Inferior longitudinal fasciculus	0.056 * (0.0032, 0.11)	0.054 (0.00041, 0.11)	0.049 (− 0.005, 0.10)	0.050 (− 0.004, 0.10)	0.048 (− 0.0057, 0.1)	0.047 (− 0.0068, 0.1)
Inferior fronto-occipital fasciculus	0.085 ** (0.031, 0.14)	0.028 (− 0.027, 0.083)	0.091 ** (0.036, 0.15)	0.029 (− 0.027, 0.085)	0.091 ** (0.037, 0.15)	0.031 (− 0.024, 0.086)
Uncinate fasciculus	0.052 * (0.0016, 0.10)	0.048 (− 0.0011, 0.097)	0.046 (− 0.0052, 0.097)	0.046 (− 0.0036, 0.096)	0.045 (− 0.0059, 0.096)	0.049 (− 0.0003, 0.098)
*Limbic system tracts*						
Cingulate gyrus part of the cingulum	0.020 (− 0.025, 0.065)	0.036 (− 0.0089, 0.081)	0.021 (− 0.025, 0.067)	0.032 (− 0.014, 0.078)	0.026 (− 0.02, 0.072)	0.038 (− 0.0073, 0.083)
Parahippocampal part of the cingulum	0.022 (− 0.021, 0.065)	0.0050 (− 0.039, 0.049)	0.017 (− 0.026, 0.060)	0.0069 (− 0.037, 0.051)	0.017 (− 0.026, 0.06)	0.0066 (− 0.037, 0.05)
*Callosal tracts*						
Forceps major	0.032 (− 0.02, 0.084)		0.031 (− 0.021, 0.083)		0.03 (− 0.022, 0.082)	
Forceps minor	0.036 (− 0.013, 0.085)		0.035 (− 0.015, 0.085)		0.036 (− 0.014, 0.086)	

Values represent the mean differences in *z*-score (95% confidence interval) of the SRT per standard deviation increase of the tract-specific MD. Stars indicate the significance level: * (*p* < 0.05), ** (*p* < 0.01), *** (*p* < 0.001). Results in bold were statistically significant after correction for multiple testing (*p* < 0.0032). Model I: adjusted for sex, age, age^2^, tract-specific WM volume, natural-log-transformed tract-specific WMH volume, ICV, and time between the hearing assessment and brain MRI acquisition. Model II: Model I and additionally adjusted for educational level, smoking behavior, alcohol consumption, systolic blood pressure, diastolic blood pressure, the use of anti-hypertensive drugs, and the presence of diabetes mellitus.*DIN*, digits in noise; *ICV*, intracranial volume; *MD*, mean diffusivity; *MRI*, magnetic resonance imaging; *SRT*, speech reception threshold; *WM*, white matter; *WMH*, white matter hyperintensity

Results using the SII as the dependent variable were comparable to those found with SRT (Appendix A.1 and A.2). Significant effects were also found with the MD of the inferior fronto-occipital fasciculus (*β* = 0.091, CI = [0.034, 0.15]) and the FA of the inferior longitudinal fasciculus (*β* = − 0.091, CI = [− 0.14, − 0.039]) in Model III. However, no significant association was found with the FA of the posterior thalamic radiation, whereas the relationship with the FA of the inferior fronto-occipital fasciculus became significant (*β* = − 0.081, CI = [− 0.13, − 0.028]).

We also conducted analyses using the SRT, stratifying the population by age with a threshold of 65 years, resulting in two groups. 963 participants were in the midlife group and 677 in the late-life group. These results are presented in Appendix B.1, B.2, B.3, B.4. As expected with reduced sample sizes, the statistical significance of the observed associations decreased. In the midlife group, the effect estimates of FA are similar to the whole population (Table 2), with a larger magnitude for the left superior longitudinal fasciculus, while the estimates with MD were considerably lower than those reported in Table 3. Among late-life participants, effect estimates for FA were similar to the total population, with larger estimates for the left posterior thalamic radiation and the inferior fronto-occipital fasciculus. Effect estimates with MD were generally slightly larger compared to the total population, especially for the inferior fronto-occipital fasciculus.

In a second sensitivity analysis, we used the tract-specific AD and RD as independent variables. The results with the SRT and SII can be found in Appendix C.1 & C.2 and A.3 & A.4, respectively. Comparable results are found between the SRT and SII. In general, RD exhibited larger effect estimates compared to AD for the tracts that resulted in significant associations with FA and MD (i.e., inferior fronto-occipital fasciculus, inferior longitudinal fasciculus, and posterior thalamic radiation).

## Discussion

This study provides evidence for a relationship between white matter microstructural integrity and speech perception in noise, independent of cardiovascular risk factors, general cognition, and hearing acuity in an aging population. Associations were found for the left inferior fronto-occipital fasciculus (IFO) and inferior longitudinal fasciculus (ILF) when the SRT was used as the outcome variable.

The significant association with the IFO aligns with our hypothesis that the strongest associations would be found between the outcome of the DIN test and tracts located in the ventral auditory processing stream, which is responsible for sound-to-meaning mapping (i.e., listening to meaningful speech) [9, 10]. The IFO, a major WM pathway connecting the parietal and occipital lobes to the inferior frontal lobe, plays an essential role in language comprehension, such as semantic processing [39–41]. Given its large anatomical size, it may also be involved in other functions, such as goal-oriented behavior [39, 42]. However, in the context of our study, the found association is most likely a reflection of the semantic processing capabilities.

While the uncinate fasciculus (UNC) is also considered part of the ventral auditory stream, no associations were identified with the UNC [9]. The UNC connects the frontal operculum and the anterior superior temporal gyrus [9]. Although it is considered part of the ventral auditory stream, evidence for an essential role of the UNC in language processing remains scarce [43, 44]. It even has been questioned whether the UNC should be regarded as part of the ventral stream [45], suggesting that it may play a supporting role in language, such as in mnemonic associations (e.g., linking names with faces and voices) and naming tasks [43, 46, 47]. The absence of an association with the UNC in this study may be attributed to its minimal involvement in language processing and that its associated functions are not assessed in the DIN test.

The study by Rigters et al. also investigated the relationship between tract-specific WM microstructural integrity and the SRT of the DIN test in the same Rotterdam Study cohort [3]. While both studies examined WM integrity and its relationship to auditory processing, Rigters et al. focused on the connection between WM microstructural integrity and hearing acuity. In contrast, our study aimed to isolate central auditory processing and studied the relation between this processing and WM microstructural integrity. Rigters et al. accounted for peripheral hearing loss using the PTA. In contrast, we excluded measurements that were likely determined by the degree of hearing loss and applied SII-modeling to isolate central auditory processing, thereby minimizing the effects of peripheral hearing abilities. While Rigters et al. identified an association between the FA of the UNC and SRT, our analysis did not support this finding. Their findings may have been influenced by audibility aspects, as they also identified a significant relationship between the MD of the UNC and the average pure-tone high frequency thresholds. This difference in results may suggest that in the study of Rigters et al., hearing acuity, rather than the speech perception abilities, was the underlying factor influencing the relationship between the FA of the UNC and the SRT. It may be inadequate to rely on the average hearing thresholds to correct for the effects of hearing acuity.

Another recent study found associations between longitudinal changes in WM microstructural integrity of the UNC and speech-in-noise perception, in which a poorer SRT was related to a faster decline in microstructural integrity, suggesting a possible causal relationship [19]. Armstrong et al. also accounted for peripheral hearing with average hearing thresholds, raising the possibility of confounding by peripheral hearing loss. The differences in findings may also be attributed to the speech material used across the studies. While we used digits in noise, Armstrong et al. used sentences from the QuickSIN test [48]. The use of sentences in speech-in-noise tests places a greater demand on cognitive resources than the use of digits, potentially introducing additional cognitive factors [18].

In recent work, we investigated the longitudinal association between peripheral hearing loss, rather than central auditory processing, and tract-specific WM microstructural integrity [49]. We found that hearing loss was associated with worse WM microstructural integrity in the left superior longitudinal fasciculus and UNC. Even with a substantial overlap in participants between the current study and this other recent work, the focus on peripheral versus central aspects of hearing led to different associations with WM tracts. The difference in findings highlights the necessity of investigating both the central and peripheral auditory systems and their relationship to brain structure. Moreover, the differences reinforce the importance of appropriate corrections for peripheral hearing loss when assessing the central auditory system, using speech-in-noise tests. Failure to do so may obscure the contribution of the central auditory system.

In the present study, we also found significant associations with the left ILF and PTR. The ILF, which is primarily involved in visual perception, is also considered to be part of the ventral auditory stream by some studies (i.e., sound-to-meaning mapping) [50–52]. Other studies have indicated that speech understanding is too complex to be solely mediated by language areas and requires an extensive left-sided network [45, 53, 54]. The ILF is considered to be one of the prominent tracts within this network, alongside the IFO [53]. While not all regions and pathways are directly involved, some may facilitate speech understanding through, for instance, working memory and cognitive control [55]. Given that the association with the ILF remained after controlling for cognition with the MMSE, it is likely that the ILF is directly involved in language processing. Therefore, we believe that the association found with the ILF is also related to central auditory processing, like with the IFO.

The PTR connects the thalamus to the occipital and parietal lobes. Its function is to relay sensory and/or motor information to these areas of the cortex, which are not related to language. However, thalamic radiations are associated with attention, which in turn affects speech understanding [56–58]. The influence of attention is most pronounced for degraded speech, such as the DIN test, because it requires more attentional resources [59]. The thalamus is also regarded as a central monitor of language-specific cortical activities [60]. Poorer microstructure in the PTR could disrupt this regulatory function. Since both possible interpretations of the significant associations with the left PTR do not suggest a direct involvement in auditory processing and the association was not significant after controlling for general cognition, we consider it to be a primarily cognitive effect.

In addition to the SRT, we used an audibility-corrected measure of speech understanding, namely the SII. The results obtained with the SII were comparable to those obtained with the SRT. This is likely attributable to the exclusion of participants with a PTA exceeding 25 dB HL, to minimize the potential influence of audibility on the SRT. However, even with a low PTA, it is still possible that audibility was not optimal in a smaller frequency range, such as the high frequencies (4–8 kHz). The SII corrects for audibility effects even in smaller frequency ranges, thus better covering central aspects of auditory processing. With the SII, no associations were found, indicating that audibility may still exert a modest role in the association found with the SRT. This association found with the PTR may be attributed to the reduced attentional resources associated with age-related hearing loss, as well as the role of the PTR in attention [56, 57, 61]. The association with MD of the left IFO became significant with the SII. This further supports the interpretation that the IFO reflects central aspects of auditory processing.

In the sensitivity analysis, we stratified the study population into two groups: a midlife and a late-life group. The threshold of 65 years was chosen based on the Lancet report, which states that the effects of hearing loss on dementia are most prominent during midlife [1]. We found that effect estimates of FA were comparable between the midlife and late-life groups. However, a stronger effect estimate was found for the superior longitudinal fasciculus in the midlife group, with larger estimates for the posterior thalamic radiation and inferior fronto-occipital fasciculus in the late-life group. These results suggest that the observed effects of FA in the total study population are likely to be driven by contributions from both age groups. For MD, effect estimates were lower for the midlife group compared to the total population and stronger for the late-life group. The difference between the findings with FA and MD may be related to their relationships with age. FA tends to decline linearly with age, while the trajectory of MD is non-linear [62, 63]. Larger changes in MD may occur later in life, resulting in larger variability among participants and potential amplification of effects found for the late-life group. Secondly, it may be plausible that the non-linearity of MD was not fully accounted for and that residual aging effects have driven the associations. Therefore, MD findings should be interpreted with a degree of caution.

The effect estimates of some white matter tracts were stronger in a specific age group, which may be attributed to the regional variation in timing of microstructural degradation [64]. Tracts that may have a later onset may only show sufficient variation between participants at a later age, possibly limiting the detection of associations among younger participants.

In the second sensitivity analysis, we found that the associations with FA and MD were primarily driven by increases in RD. Elevated RD is associated with degradation of myelin [65], which plays a role in the quick and efficient transmission of neural signals. Therefore, our findings suggest that disruptions in the signal transmission within the ILF and IFO associate with poorer speech understanding in noise.

The main rationale for this study was that central auditory functioning could serve as a marker for cognitive decline and dementia [7]. After accounting for general cognition, we only found associations with the ILF and IFO, which both have a prominent role in speech processing. This suggests that we primarily assessed the speech processing capacity. Therefore, central auditory processing may not be a reliable marker for cognitive decline. However, speech-in-noise tests that use sentences as speech material may be a valuable marker for cognitive decline, as these tests engage cognitive functions to a greater extent than the DIN test [18]. We propose that the hypothesis linking central auditory processing to cognitive decline should be adapted to suggest that speech understanding could be the actual marker. Future research should be conducted to examine whether this is a valuable marker.

We have not done any analysis on sex difference. Other studies did find differences in cognitive performance in men and women in aging populations. Therefore, it would be interesting to investigate whether there are differences in central auditory processing between men and women [66, 67].

In light of the hypothesis that an extensive left-sided brain network is recruited for speech understanding [53, 54], future research could focus on elucidating the relationship between speech understanding and structural brain connectivity. This connectivity can be reconstructed using a DTI-based approach [68]. The proposed future research could enhance our understanding of important connections within the hypothesized network for speech understanding. This could increase the knowledge of the extent to which other cognitive functions are engaged when listening to speech.

The strengths of this study are its large population-based sample, the availability of both auditory and neuroimaging assessments, exclusion of participants with hearing loss (PTA > 25 dB HL), and the use of the SII to correct for audibility effects. However, it is important to note several limitations. The outcome of the DIN test depends on attention. Participants in the Rotterdam Study are subjected to more assessments, which may have resulted in fatigue, which could subsequently have affected their attention. Second, the central aspects of auditory processing were only indirectly assessed through speech understanding. Several aspects of central auditory processing, such as binaural or dichotic processing, were not included. Tests that are more specific to central auditory would have been beneficial in this study, although it should be acknowledged that these are also influenced by cognition and peripheral hearing loss [69]. Unfortunately, additional tests were not possible in the Rotterdam Study due to time constraints. Finally, the cross-sectional design of this study precludes any causal interpretation of the results. Longitudinal studies are needed to assess a causal relationship between central auditory processing and brain health.

## Conclusions

Poor speech understanding in noise in older adults without significant hearing impairment relates to degraded WM microstructure in the left IFO and ILF. When audibility effects were taken into account, the associations remained significant, and the effect estimates were even stronger. Both tracts play a crucial role in speech perception, indicating that we primarily assessed central auditory processes involved in speech processing. The results lend support to the hypothesis that the important role of central processing in speech understanding in noise involves an extensive left-sided network, encompassing the IFO and ILF and extends beyond the language-related brain regions. We argue that with our methodology, we more effectively captured the central auditory system than previous investigations into the relationship between speech perception and WM microstructure. The results suggest that auditory function in the elderly population may be additionally affected by age-related decline in these brain regions.

## Supplementary Information

Below is the link to the electronic supplementary material.

ESM 1 (DOCX 49.8 KB)

ESM 2 (DOCX 50.8 KB)

ESM 3 (DOCX 38.9 KB)

## Data Availability

Data can be obtained upon request. Requests should be directed towards the management team of the Rotterdam Study (datamanagement.ergo@erasmusmc.nl), which has a protocol for approving data requests. Because of restrictions based on privacy regulations and informed consent of the participants, data cannot be made freely available in a public repository.
