# Supplementary material for: Loss of microstructural integrity in left hemispheric white matter tracts is associated with poorer digits in noise understanding
Source: GeroScience. 2025 May 26;48(1):1063–77. doi: 10.1007/s11357-025-01707-5 (PMC12972419; doi:10.1007/s11357-025-01707-5)
Supplement: Supplementary file 1 — (DOCX 49.8 KB) [file 11357_2025_1707_MOESM1_ESM.docx]

**Appendix A. Supplementary data**

**Table A.1**

Associations between the SII and the tract-specific FAs

| WM tracts | Model I | | Model II | | | Model III | | |
| --- | --- | --- | --- | --- | --- | --- | --- | --- |
|  | Left hemisphere | Right hemisphere | Left hemisphere | Right hemisphere | | Left hemisphere | | Right hemisphere |
| *Brainstem tracts* | | | | |  | |  | |
| Middle cerebellar peduncle | -0.025  (-0.072, 0.022) | | -0.026  (-0.074, 0.022) | | | -0.014  (-0.062, 0.034) | | |
| Medial lemniscus | 0.018  (-0.033, 0.069) | 0.017  (-0.034, 0.068) | 0.033  (-0.018, 0.084) | 0.036  (-0.015, 0.087) | | 0.036  (-0.015, 0.087) | | 0.037  (-0.014, 0.088) |
| *Projection tracts* | | | | |  | |  | |
| Corticospinal tract | 0.028  (-0.020, 0.076) | -0.00077  (-0.048, 0.047) | 0.039  (-0.0097, 0.088) | 0.011  (-0.037, 0.059) | | 0.039  (-0.0095, 0.087) | | 0.011  (-0.036, 0.058) |
| Anterior thalamic radiation | -0.034  (-0.091, 0.023) | -0.035  (-0.094, 0.024) | -0.033  (-0.091, 0.025) | -0.034  (-0.093, 0.025) | | -0.033  (-0.09, 0.024) | | -0.034  (-0.093, 0.025) |
| Superior thalamic radiation | -0.011  (-0.062, 0.040) | -0.012  (-0.062, 0.038) | -0.0082  (-0.060, 0.043) | -0.0043  (-0.054, 0.046) | | -0.011  (-0.062, 0.040) | | -0.0042  (-0.054, 0.045) |
| Posterior thalamic radiation | **-0.087 ****  **(-0.14, -0.034)** | -0.039  (-0.094, 0.016) | -0.080 **  (-0.13, -0.026) | -0.029  (-0.085, 0.027) | | -0.075 **  (-0.13, -0.022) | | -0.034  (-0.089, 0.021) |
| *Association tracts* | | | | |  | |  | |
| Superior longitudinal fasciculus | -0.051  (-0.10, 0.0015) | -0.0094  (-0.063, 0.045) | -0.051  (-0.10, 0.0022) | -0.0039  (-0.059, 0.051) | | -0.051  (-0.1, 0.0019) | | -0.0092  (-0.064, 0.045) |
| Inferior longitudinal fasciculus | **-0.10 *****  **(-0.15, -0.048)** | -0.043  (-0.094, 0.0083) | **-0.095 *****  **(-0.15, -0.043)** | -0.037  (-0.089, 0.015) | | **-0.091 *****  **(-0.14, -0.039)** | | -0.038 (  -0.09, 0.014) |
| Inferior fronto-occipital fasciculus | **-0.082 ****  **(-0.13, -0.029)** | -0.050  (-0.10, 0.0023) | **-0.083 ****  **(-0.14, -0.03)** | -0.051  (-0.10, 0.0024) | | **-0.081 ****  **(-0.13, -0.028)** | | -0.052  (-0.11, 0.0011) |
| Uncinate fasciculus | -0.057 *  (-0.11, -0.0014) | -0.013  (-0.065, 0.039) | -0.058 *  (-0.11, -0.002) | -0.014  (-0.067, 0.039) | | -0.055  (-0.11, 0.00065) | | -0.017  (-0.069, 0.035) |
| *Limbic system tracts* | | | | |  | |  | |
| Cingulate gyrus part of the cingulum | 0.021  (-0.028, 0.070) | -0.045  (-0.092, 0.0024) | 0.017  (-0.032, 0.066) | -0.04  (-0.088, 0.0075) | | 0.013  (-0.036, 0.062) | | -0.043  (-0.09, 0.0042) |
| Parahippocampal part of the cingulum | 0.0027  (-0.046, 0.051) | 0.0045  (-0.045, 0.054) | 0.010  (-0.039, 0.059) | 0.00037  (-0.049, 0.049) | | 0.011  (-0.037, 0.059) | | 0.00024  (-0.049, 0.049) |
| *Callosal tracts* | | | | |  | |  | |
| Forceps major | -0.037 (-0.094, 0.020) | | -0.032 (-0.089, 0.025) | | | -0.033 (-0.09, 0.024) | | |
| Forceps minor | -0.019 (-0.074, 0.036) | | -0.015 (-0.071, 0.041) | | | -0.015 (-0.071, 0.041) | | |

Values represent the mean differences in z-score (95% confidence interval) of the SII per standard deviation increase of the tract-specific MD. Stars indicate the significance level: * (p < 0.05), ** (p < 0.01), *** (p < 0.001). Results in bold were statistically significant after correction for multiple testing (p < 0.0032). Model I: adjusted for sex, age, age^2^, PTA, tract-specific WM volume, natural-log-transformed tract-specific WMH volume, ICV, and time between the hearing assessment and brain MRI acquisition. Model II: Model I and additionally adjusted for educational level, smoking behaviour, alcohol consumption, systolic blood pressure, diastolic blood pressure, the use of anti-hypertensive drugs and the presence of diabetes mellitus.

**Table A.2**

Associations between the SII and the tract-specific MDs

| WM tracts | | Model I | | | | Model II | | | | | Model III | | | |
| --- | --- | --- | --- | --- | --- | --- | --- | --- | --- | --- | --- | --- | --- | --- |
|  | Left hemisphere | | Right hemisphere | | Left hemisphere | | Right hemisphere | | | Left hemisphere | | | Right hemisphere | |
| *Brainstem tracts* | | | | | | | | |  | | |  | | |
| Middle cerebellar peduncle | | -0.00049  (-0.045, 0.044) | | | | -0.0061  (-0.051, 0.039) | | | | | -0.009  (-0.054, 0.036) | | | |
| Medial lemniscus | | -0.040  (-0.086, 0.0056) | | -0.018  (-0.064, 0.028) | | -0.051 *  (-0.097, -0.0052) | | -0.032  (-0.078, 0.014) | | | -0.048 *  (-0.094, -0.0024) | | | -0.027  (-0.073, 0.019) |
| *Projection tracts* | | | | | | | | |  | | |  | | |
| Corticospinal tract | | 0.0048  (-0.053, 0.063) | | 0.074  (0.017, 0.13) | | -0.0043  (-0.063, 0.055) | | 0.062  (0.0046, 0.12) | | | 0.0012  (-0.058, 0.06) | | | 0.065 *  (0.0078, 0.12) |
| Anterior thalamic radiation | | 0.050  (-0.024, 0.12) | | 0.037  (-0.035, 0.11) | | 0.039  (-0.036, 0.11) | | 0.031  (-0.042, 0.1) | | | 0.039  (-0.035, 0.11) | | | 0.027  (-0.045, 0.099) |
| Superior thalamic radiation | | 0.073 *  (0.011, 0.13) | | 0.061  (0.0012, 0.12) | | 0.069 *  (0.0056, 0.13) | | 0.053  (-0.0076, 0.11) | | | 0.072 *  (0.009, 0.14) | | | 0.049  (-0.011, 0.11) |
| Posterior thalamic radiation | | 0.056 *  (0.0022, 0.11) | | 0.035  (-0.020, 0.090) | | 0.059 *  (0.0052, 0.11) | | 0.033  (-0.022, 0.088) | | | 0.058 *  (0.0045, 0.11) | | | 0.028  (-0.027, 0.083) |
| *Association tracts* | | | | | | | | |  | | |  | | |
| Superior longitudinal fasciculus | | 0.047  (-0.0098, 0.1) | | 0.015  (-0.042, 0.072) | | 0.045  (-0.013, 0.1) | | 0.014  (-0.043, 0.071) | | | 0.047  (-0.01, 0.10) | | | 0.017  (-0.04, 0.074) |
| Inferior longitudinal fasciculus | | 0.057 *  (0.0015, 0.11) | | 0.055  (-0.0014, 0.11) | | 0.050  (-0.0066, 0.11) | | 0.050  (-0.0067, 0.11) | | | 0.049  (-0.0072, 0.11) | | | 0.048  (-0.0083, 0.10) |
| Inferior fronto-occipital fasciculus | | 0.086 **  (0.029, 0.14) | | 0.023  (-0.034, 0.08) | | **0.091 ****  **(0.034, 0.15)** | | 0.025  (-0.033, 0.083) | | | **0.091 ****  **(0.034, 0.15)** | | | 0.027  (-0.031, 0.085) |
| Uncinate fasciculus | | 0.057 *  (0.004, 0.11) | | 0.047  (-0.0047, 0.099) | | 0.049  (-0.0047, 0.1) | | 0.043  (-0.0091, 0.095) | | | 0.048  (-0.0054, 0.10) | | | 0.046  (-0.0058, 0.098) |
| *Limbic system tracts* | | | | | | | | |  | | |  | | |
| Cingulate gyrus part of the cingulum | | 0.017  (-0.031, 0.065) | | 0.040  (-0.0072, 0.087) | | 0.017  (-0.031, 0.065) | | 0.033  (-0.015, 0.081) | | | 0.022  (-0.026, 0.070) | | | 0.039  (-0.0085, 0.086) |
| Parahippocampal part of the cingulum | | 0.021  (-0.024, 0.066) | | 0.0063  (-0.040, 0.052) | | 0.017  (-0.028, 0.062) | | 0.0070  (-0.039, 0.053) | | | 0.016  (-0.029, 0.061) | | | 0.0071  (-0.038, 0.053) |
| *Callosal tracts* | | | | | | | | |  | | |  | | |
| Forceps major | | 0.021 (-0.033, 0.075) | | | | 0.019 (-0.036, 0.074) | | | | | 0.018 (-0.036, 0.072) | | | |
| Forceps minor | | 0.033 (-0.019, 0.085) | | | | 0.031 (-0.022, 0.084) | | | | | 0.033 (-0.019, 0.085) | | | |

Values represent the mean differences in z-score (95% confidence interval) of the SII per standard deviation increase of the tract-specific FA. Stars indicate the significance level: * (p < 0.05), ** (p < 0.01), *** (p < 0.001). Results in bold were statistically significant after correction for multiple testing (p < 0.0032). Model I: adjusted for sex, age, age^2^, PTA, tract-specific WM volume, natural-log-transformed tract-specific WMH volume, ICV, and time between the hearing assessment and brain MRI acquisition. Model II: Model I and additionally adjusted for educational level, smoking behaviour, alcohol consumption, systolic blood pressure, diastolic blood pressure, the use of anti-hypertensive drugs and the presence of diabetes mellitus.

**Table A.3**

Associations between the SII and the tract-specific ADs

| WM tracts | | Model I | | | | Model II | | | | | Model III | | | |
| --- | --- | --- | --- | --- | --- | --- | --- | --- | --- | --- | --- | --- | --- | --- |
|  | Left hemisphere | | Right hemisphere | | Left hemisphere | | Right hemisphere | | | Left hemisphere | | | Right hemisphere | |
| *Brainstem tracts* | | | | | | | | |  | | |  | | |
| Middle cerebellar peduncle | | -0.015  (-0.063, 0.033) | | | | -0.021  (-0.069, 0.027) | | | | | -0.019  (-0.067, 0.029) | | | |
| Medial lemniscus | | -0.028  (-0.078, 0.022) | | -0.020  (-0.07, 0.030) | | -0.028  (-0.078, 0.022) | | -0.020  (-0.07, 0.030) | | | -0.023  (-0.073, 0.027) | | | -0.015  (-0.065, 0.035) |
| *Projection tracts* | | | | | | | | |  | | |  | | |
| Corticospinal tract | | 0.052  (-0.006, 0.11) | | 0.082 **  (0.024, 0.14) | | 0.054  (-0.0047, 0.11) | | 0.081 **  (0.023, 0.14) | | | 0.059 *  (0.00049, 0.12) | | | 0.082 **  (0.024, 0.14) |
| Anterior thalamic radiation | | 0.033  (-0.040, 0.11) | | 0.023  (-0.048, 0.094) | | 0.021  (-0.052, 0.094) | | 0.019  (-0.052, 0.09) | | | 0.020  (-0.052, 0.092) | | | 0.016  (-0.055, 0.087) |
| Superior thalamic radiation | | 0.090 **  (0.029, 0.15) | | 0.084 **  (0.023, 0.15) | | 0.084 **  (0.022, 0.15) | | 0.081 *  (0.019, 0.14) | | | 0.084 **  (0.022, 0.15) | | | 0.078 *  (0.017, 0.14) |
| Posterior thalamic radiation | | 0.046  (-0.011, 0.1) | | 0.024  (-0.032, 0.080) | | 0.051  (-0.0054, 0.11) | | 0.025  (-0.031, 0.081) | | | 0.054  (-0.002, 0.11) | | | 0.019  (-0.037, 0.075) |
| *Association tracts* | | | | | | | | |  | | |  | | |
| Superior longitudinal fasciculus | | 0.032  (-0.026, 0.090) | | 0.023  (-0.037, 0.083) | | 0.028  (-0.030, 0.086) | | 0.025  (-0.035, 0.085) | | | 0.031  (-0.027, 0.089) | | | 0.027  (-0.032, 0.086) |
| Inferior longitudinal fasciculus | | 0.015  (-0.041, 0.071) | | 0.044  (-0.013, 0.10) | | 0.014  (-0.043, 0.071) | | 0.045  (-0.012, 0.10) | | | 0.014  (-0.042, 0.070) | | | 0.043  (-0.014, 0.10) |
| Inferior fronto-occipital fasciculus | | 0.067 *  (0.010, 0.12) | | -0.0022  (-0.058, 0.054) | | 0.073 *  (0.016, 0.13) | | -0.0024  (-0.059, 0.054) | | | 0.075 **  (0.018, 0.13) | | | -0.00049  (-0.056, 0.056) |
| Uncinate fasciculus | | 0.057 *  (0.0021, 0.11) | | 0.059 *  (0.0057, 0.11) | | 0.043  (-0.012, 0.098) | | 0.052  (-0.0015, 0.11) | | | 0.044  (-0.011, 0.099) | | | 0.055 *  (0.0018, 0.11) |
| *Limbic system tracts* | | | | | | | | |  | | |  | | |
| Cingulate gyrus part of the cingulum | | 0.030  (-0.02, 0.08) | | -0.020  (-0.069, 0.029) | | 0.027  (-0.023, 0.077) | | -0.022  (-0.071, 0.027) | | | 0.027  (-0.022, 0.076) | | | -0.020  (-0.069, 0.029) |
| Parahippocampal part of the cingulum | | 0.036  (-0.012, 0.084) | | 0.016  (-0.033, 0.065) | | 0.040  (-0.0086, 0.089) | | 0.015  (-0.034, 0.064) | | | 0.039  (-0.0093, 0.087) | | | 0.015  (-0.034, 0.064) |
| *Callosal tracts* | | | | | | | | |  | | |  | | |
| Forceps major | | 0.013 (-0.039, 0.065) | | | | 0.013 (-0.039, 0.065) | | | | | 0.011 (-0.041, 0.063) | | | |
| Forceps minor | | 0.015 (-0.036, 0.066) | | | | 0.016 (-0.035, 0.067) | | | | | 0.018 (-0.032, 0.068) | | | |

Values represent the mean differences in z-score (95% confidence interval) of the SII per standard deviation increase of the tract-specific AD. Stars indicate the significance level: * (p < 0.05), ** (p < 0.01), *** (p < 0.001). Results in bold were statistically significant after correction for multiple testing (p < 0.0032). Model I: adjusted for sex, age, age^2^, PTA, tract-specific WM volume, natural-log-transformed tract-specific WMH volume, ICV, and time between the hearing assessment and brain MRI acquisition. Model II: Model I and additionally adjusted for educational level, smoking behaviour, alcohol consumption, systolic blood pressure, diastolic blood pressure, the use of anti-hypertensive drugs and the presence of diabetes mellitus.

**Table A.4**

Associations between the SII and the tract-specific RDs

| WM tracts | | Model I | | | | Model II | | | | | Model III | | | |
| --- | --- | --- | --- | --- | --- | --- | --- | --- | --- | --- | --- | --- | --- | --- |
|  | Left hemisphere | | Right hemisphere | | Left hemisphere | | Right hemisphere | | | Left hemisphere | | | Right hemisphere | |
| *Brainstem tracts* | | | | | | | | |  | | |  | | |
| Middle cerebellar peduncle | | -0.0038  (-0.051, 0.043) | | | | -0.0048  (-0.052, 0.043) | | | | | -0.0095  (-0.057, 0.038) | | | |
| Medial lemniscus | | -0.043  (-0.092, 0.0064) | | -0.018  (-0.068, 0.032) | | -0.060 *  (-0.11, -0.011) | | -0.037  (-0.087, 0.013) | | | -0.057 *  (-0.11, -0.0079) | | | -0.033  (-0.083, 0.017) |
| *Projection tracts* | | | | | | | | |  | | |  | | |
| Corticospinal tract | | -0.022  (-0.078, 0.034) | | 0.040  (-0.015, 0.095) | | -0.035  (-0.092, 0.022) | | 0.025  (-0.030, 0.080) | | | -0.031  (-0.088, 0.026) | | | 0.025  (-0.030, 0.080) |
| Anterior thalamic radiation | | 0.048  (-0.029, 0.12) | | 0.048  (-0.028, 0.12) | | 0.038  (-0.040, 0.12) | | 0.044  (-0.033, 0.12) | | | 0.036  (-0.041, 0.11) | | | 0.039  (-0.038, 0.12) |
| Superior thalamic radiation | | 0.037  (-0.025, 0.099) | | 0.054  (-0.0056, 0.11) | | 0.033  (-0.030, 0.096) | | 0.046  (-0.014, 0.11) | | | 0.037  (-0.026, 0.10) | | | 0.042  (-0.018, 0.10) |
| Posterior thalamic radiation | | 0.080 **  (0.023, 0.14) | | 0.039  (-0.020, 0.098) | | 0.080 **  (0.023, 0.14) | | 0.036  (-0.023, 0.095) | | | 0.078 **  (0.022, 0.13) | | | 0.034  (-0.025, 0.093) |
| *Association tracts* | | | | | | | | |  | | |  | | |
| Superior longitudinal fasciculus | | 0.056  (-0.0034, 0.12) | | 0.019  (-0.041, 0.079) | | 0.053  (-0.0071, 0.11) | | 0.015  (-0.045, 0.075) | | | 0.055  (-0.0048, 0.11) | | | 0.018  (-0.042, 0.078) |
| Inferior longitudinal fasciculus | | 0.083 **  (0.024, 0.14) | | 0.062 *  (0.003, 0.12) | | 0.075 *  (0.015, 0.13) | | 0.057  (-0.0023, 0.12) | | | 0.073 *  (0.014, 0.13) | | | 0.055  (-0.004, 0.11) |
| Inferior fronto-occipital fasciculus | | **0.11 *****  **(0.049, 0.17)** | | 0.032  (-0.029, 0.093) | | **0.12 *****  **(0.059, 0.18)** | | 0.034  (-0.028, 0.096) | | | **0.12 *****  **(0.06, 0.18)** | | | 0.037  (-0.025, 0.099) |
| Uncinate fasciculus | | 0.070 *  (0.013, 0.13) | | 0.047  (-0.0081, 0.10) | | 0.065  (0.0073, 0.12) | | 0.044  (-0.011, 0.099) | | | 0.062  (0.0047, 0.12) | | | 0.047  (-0.0082, 0.1) |
| *Limbic system tracts* | | | | | | | | |  | | |  | | |
| Cingulate gyrus part of the cingulum | | -0.0075  (-0.059, 0.044) | | 0.059 *  (0.0089, 0.11) | | -0.0058  (-0.058, 0.047) | | 0.054 *  (0.0036, 0.10) | | | 0.00  (-0.052, 0.052) | | | 0.059 *  (0.0089, 0.11) |
| Parahippocampal part of the cingulum | | 0.027  (-0.021, 0.075) | | 0.012  (-0.037, 0.061) | | 0.025  (-0.023, 0.073) | | 0.014  (-0.035, 0.063) | | | 0.024  (-0.023, 0.071) | | | 0.014  (-0.035, 0.063) |
| *Callosal tracts* | | | | | | | | |  | | |  | | |
| Forceps major | | 0.039 (-0.020, 0.098) | | | | 0.039 (-0.020, 0.098) | | | | | 0.039 (-0.020, 0.098) | | | |
| Forceps minor | | 0.041 (-0.016, 0.098) | | | | 0.038 (-0.020, 0.096) | | | | | 0.038 (-0.020, 0.096) | | | |

Values represent the mean differences in z-score (95% confidence interval) of the SII per standard deviation increase of the tract-specific RD. Stars indicate the significance level: * (p < 0.05), ** (p < 0.01), *** (p < 0.001). Results in bold were statistically significant after correction for multiple testing (p < 0.0032). Model I: adjusted for sex, age, age^2^, PTA, tract-specific WM volume, natural-log-transformed tract-specific WMH volume, ICV, and time between the hearing assessment and brain MRI acquisition. Model II: Model I and additionally adjusted for educational level, smoking behaviour, alcohol consumption, systolic blood pressure, diastolic blood pressure, the use of anti-hypertensive drugs and the presence of diabetes mellitus.
