# Supplementary material for: Loss of microstructural integrity in left hemispheric white matter tracts is associated with poorer digits in noise understanding
Source: GeroScience. 2025 May 26;48(1):1063–77. doi: 10.1007/s11357-025-01707-5 (PMC12972419; doi:10.1007/s11357-025-01707-5)
Supplement: Supplementary file 2 — (DOCX 50.8 KB) [file 11357_2025_1707_MOESM2_ESM.docx]

**Appendix B. Supplementary data**

**Table B.1**

Associations between the SRT and the tract-specific FAs for participants younger than 65 years (n = 993)

| WM tracts | | Model I | | | | Model II | | | | | Model III | | | |
| --- | --- | --- | --- | --- | --- | --- | --- | --- | --- | --- | --- | --- | --- | --- |
|  | Left hemisphere | | Right hemisphere | | Left hemisphere | | Right hemisphere | | | Left hemisphere | | | Right hemisphere | |
| *Brainstem tracts* | | | | | | | | |  | | |  | | |
| Middle cerebellar peduncle | | -0.047 (-0.11, 0.011) | | | | -0.058 (-0.12, 0.00097) | | | | | -0.047 (-0.11, 0.012) | | | |
| Medial lemniscus | | -0.026  (-0.091, 0.039) | | -0.017  (-0.082, 0.048) | | -0.012  (-0.076, 0.052) | | -0.0041 (-0.068, 0.060) | | | -0.012 (-0.076, 0.052) | | | -0.0062 (-0.070, 0.058) |
| *Projection tracts* | | | | | | | | |  | | |  | | |
| Corticospinal tract | | 0.0074  (-0.053, 0.068) | | -0.019  (-0.077, 0.039) | | 0.016  (-0.044, 0.076) | | -0.0082  (-0.067, 0.05) | | | 0.012  (-0.048, 0.072) | | | -0.012  (-0.070, 0.046) |
| Anterior thalamic radiation | | -0.032  (-0.10, 0.038) | | -0.028  (-0.10, 0.044) | | -0.030  (-0.10, 0.040) | | -0.029  (-0.10, 0.042) | | | -0.032  (-0.10, 0.038) | | | -0.029  (-0.10, 0.042) |
| Superior thalamic radiation | | -0.029  (-0.092, 0.034) | | -0.010  (-0.073, 0.053) | | -0.030  (-0.093, 0.033) | | -0.0030  (-0.065, 0.059) | | | -0.036  (-0.099, 0.027) | | | -0.00062  (-0.063, 0.061) |
| Posterior thalamic radiation | | -0.066 *  (-0.13, -0.0024) | | -0.013  (-0.08, 0.054) | | -0.046  (-0.11, 0.017) | | -0.0058  (-0.072, 0.06) | | | -0.04  (-0.10, 0.023) | | | -0.0055  (-0.071, 0.06) |
| *Association tracts* | | | | | | | | |  | | |  | | |
| Superior longitudinal fasciculus | | -0.073 *  (-0.14, -0.011) | | -0.012  (-0.079, 0.055) | | -0.069 *  (-0.13, -0.0068) | | -0.0034  (-0.070, 0.063) | | | -0.070 *  (-0.13, -0.0082) | | | -0.0083  (-0.074, 0.058) |
| Inferior longitudinal fasciculus | | **-0.098 ****  **(-0.16, -0.036)** | | -0.0015  (-0.065, 0.062) | | -0.086 **  (-0.15, -0.024) | | 0.0076  (-0.056, 0.071) | | | -0.081 *  (-0.14, -0.019) | | | 0.011  (-0.052, 0.074) |
| Inferior fronto-occipital fasciculus | | -0.057  (-0.12, 0.0064) | | -0.037  (-0.099, 0.025) | | -0.053  (-0.12, 0.010) | | -0.033  (-0.096, 0.03) | | | -0.048  (-0.11, 0.015) | | | -0.032  (-0.094, 0.03) |
| Uncinate fasciculus | | -0.044  (-0.11, 0.024) | | 0.0077  (-0.054, 0.070) | | -0.038  (-0.11, 0.030) | | 0.012  (-0.049, 0.073) | | | -0.036  (-0.10, 0.031) | | | 0.0091  (-0.052, 0.070) |
| *Limbic system tracts* | | | | | | | | |  | | |  | | |
| Cingulate gyrus part of the cingulum | | 0.045 (-0.014, 0.10) | | -0.026  (-0.084, 0.032) | | 0.037  (-0.022, 0.096) | | -0.026  (-0.084, 0.032) | | | 0.033  (-0.026, 0.092) | | | -0.027  (-0.084, 0.03) |
| Parahippocampal part of the cingulum | | 0.024 (-0.036, 0.084) | | 0.014  (-0.047, 0.075) | | 0.030  (-0.029, 0.089) | | 0.014  (-0.046, 0.074) | | | 0.033  (-0.026, 0.092) | | | 0.016  (-0.043, 0.075) |
| *Callosal tracts* | | | | | | | | |  | | |  | | |
| Forceps major | | -0.013 (-0.081, 0.055) | | | | -0.0026 (-0.07, 0.065) | | | | | -0.011 (-0.078, 0.056) | | | |
| Forceps minor | | -0.023 (-0.086, 0.04) | | | | -0.015 (-0.077, 0.047) | | | | | -0.015 (-0.077, 0.047) | | | |

Values represent the mean differences in z-score (95% confidence interval) of the SRT per standard deviation increase of the tract-specific AD. Stars indicate the significance level: * (p < 0.05), ** (p < 0.01), *** (p < 0.001). Results in bold were statistically significant after correction for multiple testing (p < 0.0032). Model I: adjusted for sex, age, age^2^, PTA, tract-specific WM volume, natural-log-transformed tract-specific WMH volume, ICV, and time between the hearing assessment and brain MRI acquisition. Model II: Model I and additionally adjusted for educational level, smoking behaviour, alcohol consumption, systolic blood pressure, diastolic blood pressure, the use of anti-hypertensive drugs and the presence of diabetes mellitus.

**Table B.2**

Associations between the SRT and the tract-specific MDs for participants younger than 65 years (n = 993)

| WM tracts | | Model I | | | | Model II | | | | | Model III | | | |
| --- | --- | --- | --- | --- | --- | --- | --- | --- | --- | --- | --- | --- | --- | --- |
|  | Left hemisphere | | Right hemisphere | | Left hemisphere | | Right hemisphere | | | Left hemisphere | | | Right hemisphere | |
| *Brainstem tracts* | | | | | | | | |  | | |  | | |
| Middle cerebellar peduncle | | -0.0018 (-0.057, 0.054) | | | | -0.017 (-0.073, 0.039) | | | | | -0.018 (-0.073, 0.037) | | | |
| Medial lemniscus | | -0.042  (-0.098, 0.014) | | -0.012  (-0.070, 0.046) | | -0.047  (-0.10, 0.009) | | -0.024  (-0.082, 0.034) | | | -0.040  (-0.096, 0.016) | | | -0.018  (-0.076, 0.040) |
| *Projection tracts* | | | | | | | | |  | | |  | | |
| Corticospinal tract | | 0.0075  (-0.060, 0.075) | | 0.082 *  (0.018, 0.15) | | 0.015  (-0.052, 0.082) | | 0.082 *  (0.019, 0.15) | | | 0.019  (-0.048, 0.086) | | | 0.084 **  (0.021, 0.15) |
| Anterior thalamic radiation | | 0.045  (-0.033, 0.12) | | 0.055  (-0.023, 0.13) | | 0.039  (-0.039, 0.12) | | 0.055  (-0.022, 0.13) | | | 0.035  (-0.042, 0.11) | | | 0.049  (-0.028, 0.13) |
| Superior thalamic radiation | | 0.067  (-0.002, 0.14) | | 0.056  (-0.014, 0.13) | | 0.071 *  (0.0019, 0.14) | | 0.056  (-0.014, 0.13) | | | 0.071 *  (0.0024, 0.14) | | | 0.049  (-0.020, 0.12) |
| Posterior thalamic radiation | | 0.038  (-0.025, 0.10) | | -0.014  (-0.083, 0.055) | | 0.043  (-0.019, 0.11) | | -0.0072  (-0.076, 0.061) | | | 0.041  (-0.021, 0.10) | | | -0.013  (-0.081, 0.055) |
| *Association tracts* | | | | | | | | |  | | |  | | |
| Superior longitudinal fasciculus | | 0.055  (-0.010, 0.12) | | 0.020  (-0.049, 0.089) | | 0.056  (-0.0091, 0.12) | | 0.018  (-0.051, 0.087) | | | 0.058  (-0.0067, 0.12) | | | 0.020  (-0.048, 0.088) |
| Inferior longitudinal fasciculus | | 0.022  (-0.042, 0.086) | | 8e-04  (-0.068, 0.070) | | 0.028  (-0.036, 0.092) | | -0.0024  (-0.071, 0.066) | | | 0.026  (-0.038, 0.090) | | | -0.0034  (-0.071, 0.064) |
| Inferior fronto-occipital fasciculus | | 0.047  (-0.018, 0.11) | | 0.021  (-0.046, 0.088) | | 0.049  (-0.016, 0.11) | | 0.029  (-0.038, 0.096) | | | 0.049  (-0.015, 0.11) | | | 0.033  (-0.034, 0.10) |
| Uncinate fasciculus | | 0.039  (-0.022, 0.10) | | 0.029  (-0.032, 0.090) | | 0.027  (-0.034, 0.088) | | 0.025  (-0.036, 0.086) | | | 0.026  (-0.034, 0.086) | | | 0.025  (-0.035, 0.085) |
| *Limbic system tracts* | | | | | | | | |  | | |  | | |
| Cingulate gyrus part of the cingulum | | -0.0082  (-0.066, 0.050) | | 0.018  (-0.039, 0.075) | | -0.0058  (-0.064, 0.053) | | 0.010  (-0.047, 0.067) | | | 0.00  (-0.058, 0.058) | | | 0.013  (-0.044, 0.070) |
| Parahippocampal part of the cingulum | | 0.013  (-0.042, 0.068) | | 0.009  (-0.047, 0.065) | | 0.010  (-0.045, 0.065) | | 0.017  (-0.038, 0.072) | | | 0.012  (-0.042, 0.066) | | | 0.015  (-0.040, 0.070) |
| *Callosal tracts* | | | | | | | | |  | | |  | | |
| Forceps major | | -0.031 (-0.096, 0.034) | | | | -0.039 (-0.10, 0.026) | | | | | -0.036 (-0.10, 0.028) | | | |
| Forceps minor | | 0.015 (-0.047, 0.077) | | | | 0.0086 (-0.053, 0.070) | | | | | 0.011 (-0.05, 0.072) | | | |

Values represent the mean differences in z-score (95% confidence interval) of the SRT per standard deviation increase of the tract-specific RD. Stars indicate the significance level: * (p < 0.05), ** (p < 0.01), *** (p < 0.001). Results in bold were statistically significant after correction for multiple testing (p < 0.0032). Model I: adjusted for sex, age, age^2^, PTA, tract-specific WM volume, natural-log-transformed tract-specific WMH volume, ICV, and time between the hearing assessment and brain MRI acquisition. Model II: Model I and additionally adjusted for educational level, smoking behaviour, alcohol consumption, systolic blood pressure, diastolic blood pressure, the use of anti-hypertensive drugs and the presence of diabetes mellitus.

**Table B.3**

Associations between the SRT and the tract-specific FAs for participants 65 years of age or older (n = 677)

| WM tracts | | Model I | | | | Model II | | | | | Model III | | | |
| --- | --- | --- | --- | --- | --- | --- | --- | --- | --- | --- | --- | --- | --- | --- |
|  | Left hemisphere | | Right hemisphere | | Left hemisphere | | Right hemisphere | | | Left hemisphere | | | Right hemisphere | |
| *Brainstem tracts* | | | | | | | | |  | | |  | | |
| Middle cerebellar peduncle | | -0.0063 (-0.078, 0.065) | | | | 0.0077 (-0.067, 0.082) | | | | | 0.018 (-0.057, 0.093) | | | |
| Medial lemniscus | | 0.039  (-0.035, 0.11) | | 0.033  (-0.040, 0.11) | | 0.045  (-0.030, 0.12) | | 0.043  (-0.032, 0.12) | | | 0.050  (-0.025, 0.13) | | | 0.059  (-0.026, 0.12) |
| *Projection tracts* | | | | | | | | |  | | |  | | |
| Corticospinal tract | | 0.051  (-0.023, 0.13) | | 0.011  (-0.062, 0.084) | | 0.061  (-0.017, 0.14) | | 0.017  (-0.058, 0.092) | | | 0.061  (-0.017, 0.14) | | | 0.021  (-0.054, 0.096) |
| Anterior thalamic radiation | | -0.027  (-0.11, 0.059) | | -0.031  (-0.12, 0.059) | | -0.031  (-0.12, 0.058) | | -0.035  (-0.13, 0.060) | | | -0.03  (-0.12, 0.059) | | | -0.035  (-0.13, 0.059) |
| Superior thalamic radiation | | 0.011  (-0.067, 0.089) | | -0.0059  (-0.081, 0.069) | | 0.012  (-0.070, 0.094) | | -0.0057  (-0.084, 0.072) | | | 0.0094  (-0.072, 0.091) | | | -0.0089  (-0.086, 0.069) |
| Posterior thalamic radiation | | -0.11 *  (-0.19, -0.026) | | -0.073  (-0.16, 0.013) | | -0.12 **  (-0.21, -0.033) | | -0.068  (-0.16, 0.022) | | | -0.12 **  (-0.21, -0.033) | | | -0.079  (-0.17, 0.011) |
| *Association tracts* | | | | | | | | |  | | |  | | |
| Superior longitudinal fasciculus | | -0.020  (-0.10, 0.060) | | 0.014  (-0.068, 0.096) | | -0.028  (-0.11, 0.057) | | 0.0067  (-0.079, 0.093) | | | -0.030  (-0.11, 0.054) | | | 0.0009  (-0.085, 0.087) |
| Inferior longitudinal fasciculus | | -0.098 *  (-0.18, -0.017) | | -0.086 *  (-0.16, -0.0098) | | -0.098 *  (-0.18, -0.013) | | -0.093 *  (-0.17, -0.013) | | | -0.097 *  (-0.18, -0.012) | | | -0.098 *  (-0.18, -0.018) |
| Inferior fronto-occipital fasciculus | | -0.10 *  (-0.18, -0.020) | | -0.065  (-0.15, 0.015) | | -0.12 **  (-0.20, -0.036) | | -0.080  (-0.16, 0.0049) | | | -0.12 **  (-0.20, -0.036) | | | -0.083  (-0.17, 0.0016) |
| Uncinate fasciculus | | -0.079  (-0.16, 0.0041) | | -0.043  (-0.12, 0.038) | | -0.090 *  (-0.18, -0.0032) | | -0.059  (-0.14, 0.026) | | | -0.084  (-0.17, 0.0027) | | | -0.060  (-0.14, 0.025) |
| *Limbic system tracts* | | | | | | | | |  | | |  | | |
| Cingulate gyrus part of the cingulum | | -0.022  (-0.096, 0.052) | | -0.057  (-0.13, 0.016) | | -0.025  (-0.10, 0.052) | | -0.053  (-0.13, 0.023) | | | -0.030  (-0.11, 0.047) | | | -0.058  (-0.13, 0.017) |
| Parahippocampal part of the cingulum | | -0.0036  (-0.077, 0.07) | | 0.012  (-0.062, 0.086) | | 0.005  (-0.071, 0.081) | | -0.00062  (-0.077, 0.076) | | | 0.0031  (-0.072, 0.078) | | | -0.0019  (-0.079, 0.075) |
| *Callosal tracts* | | | | | | | | |  | | |  | | |
| Forceps major | | -0.085 (-0.17, 0.0041) | | | | -0.084 (-0.18, 0.008) | | | | | -0.080 (-0.17, 0.012) | | | |
| Forceps minor | | 0.022 (-0.063, 0.11) | | | | 0.022 (-0.066, 0.11) | | | | | 0.020 (-0.068, 0.11) | | | |

Values represent the mean differences in z-score (95% confidence interval) of the SRT per standard deviation increase of the tract-specific AD. Stars indicate the significance level: * (p < 0.05), ** (p < 0.01), *** (p < 0.001). Results in bold were statistically significant after correction for multiple testing (p < 0.0032). Model I: adjusted for sex, age, age^2^, PTA, tract-specific WM volume, natural-log-transformed tract-specific WMH volume, ICV, and time between the hearing assessment and brain MRI acquisition. Model II: Model I and additionally adjusted for educational level, smoking behaviour, alcohol consumption, systolic blood pressure, diastolic blood pressure, the use of anti-hypertensive drugs and the presence of diabetes mellitus.

**Table B.4**

Associations between the SRT and the tract-specific MDs for participants 65 years of age or older (n = 677)

| WM tracts | | Model I | | | | Model II | | | | | Model III | | | |
| --- | --- | --- | --- | --- | --- | --- | --- | --- | --- | --- | --- | --- | --- | --- |
|  | Left hemisphere | | Right hemisphere | | Left hemisphere | | Right hemisphere | | | Left hemisphere | | | Right hemisphere | |
| *Brainstem tracts* | | | | | | | | |  | | |  | | |
| Middle cerebellar peduncle | | 0.019 (-0.049, 0.087) | | | | 0.015 (-0.056, 0.086) | | | | | 0.013 (-0.057, 0.083) | | | |
| Medial lemniscus | | -0.032  (-0.10, 0.038) | | -0.023  (-0.093, 0.047) | | -0.044  (-0.12, 0.028) | | -0.025  (-0.096, 0.046) | | | -0.042  (-0.11, 0.03) | | | -0.021  (-0.092, 0.050) |
| *Projection tracts* | | | | | | | | |  | | |  | | |
| Corticospinal tract | | -0.0076  (-0.095, 0.080) | | 0.049  (-0.037, 0.13) | | -0.020  (-0.11, 0.072) | | 0.036  (-0.054, 0.13) | | | -0.015  (-0.11, 0.077) | | | 0.038  (-0.052, 0.13) |
| Anterior thalamic radiation | | 0.051  (-0.063, 0.17) | | -0.0043  (-0.12, 0.11) | | 0.042  (-0.077, 0.16) | | -0.016  (-0.14, 0.11) | | | 0.045  (-0.073, 0.16) | | | -0.014  (-0.14, 0.11) |
| Superior thalamic radiation | | 0.061  (-0.036, 0.16) | | 0.054  (-0.040, 0.15) | | 0.060  (-0.043, 0.16) | | 0.050  (-0.049, 0.15) | | | 0.066  (-0.037, 0.17) | | | 0.052  (-0.047, 0.15) |
| Posterior thalamic radiation | | 0.076  (-0.0045, 0.16) | | 0.097 *  (0.016, 0.18) | | 0.072  (-0.010, 0.15) | | 0.080  (-0.0039, 0.16) | | | 0.073  (-0.0090, 0.16) | | | 0.080  (-0.0036, 0.16) |
| *Association tracts* | | | | | | | | |  | | |  | | |
| Superior longitudinal fasciculus | | 0.038  (-0.048, 0.12) | | 0.0031  (-0.082, 0.088) | | 0.042  (-0.048, 0.13) | | 0.0089  (-0.079, 0.097) | | | 0.043  (-0.047, 0.13) | | | 0.014  (-0.074, 0.10) |
| Inferior longitudinal fasciculus | | 0.085 *  (0.00031, 0.17) | | 0.11 **  (0.027, 0.19) | | 0.075  (-0.015, 0.17) | | 0.11 *  (0.024, 0.20) | | | 0.074  (-0.016, 0.16) | | | 0.11 *  (0.024, 0.20) |
| Inferior fronto-occipital fasciculus | | **0.13 ****  **(0.045, 0.21)** | | 0.034  (-0.052, 0.12) | | **0.14 ****  **(0.053, 0.23)** | | 0.028  (-0.061, 0.12) | | | **0.14 ****  **(0.053, 0.23)** | | | 0.028  (-0.061, 0.12) |
| Uncinate fasciculus | | 0.070  (-0.0074, 0.15) | | 0.068  (-0.0079, 0.14) | | 0.071  (-0.010, 0.15) | | 0.070  (-0.0086, 0.15) | | | 0.070  (-0.011, 0.15) | | | 0.076  (-0.0024, 0.15) |
| *Limbic system tracts* | | | | | | | | |  | | |  | | |
| Cingulate gyrus part of the cingulum | | 0.054  (-0.017, 0.13) | | 0.054  (-0.017, 0.13) | | 0.056  (-0.018, 0.13) | | 0.056  (-0.018, 0.13) | | | 0.059  (-0.015, 0.13) | | | 0.064  (-0.0097, 0.14) |
| Parahippocampal part of the cingulum | | 0.036  (-0.034, 0.11) | | -0.0046  (-0.075, 0.066) | | 0.029  (-0.043, 0.10) | | -0.010  (-0.083, 0.063) | | | 0.027  (-0.045, 0.099) | | | -0.0094  (-0.082, 0.063) |
| *Callosal tracts* | | | | | | | | |  | | |  | | |
| Forceps major | | 0.10 (0.016, 0.18) * | | | | 0.10 (0.013, 0.19) * | | | | | 0.099 (0.012, 0.19) * | | | |
| Forceps minor | | 0.049 (-0.028, 0.13) | | | | 0.055 (-0.026, 0.14) | | | | | 0.059 (-0.022, 0.14) | | | |

Values represent the mean differences in z-score (95% confidence interval) of the SRT per standard deviation increase of the tract-specific RD. Stars indicate the significance level: * (p < 0.05), ** (p < 0.01), *** (p < 0.001). Results in bold were statistically significant after correction for multiple testing (p < 0.0032). Model I: adjusted for sex, age, age^2^, PTA, tract-specific WM volume, natural-log-transformed tract-specific WMH volume, ICV, and time between the hearing assessment and brain MRI acquisition. Model II: Model I and additionally adjusted for educational level, smoking behaviour, alcohol consumption, systolic blood pressure, diastolic blood pressure, the use of anti-hypertensive drugs and the presence of diabetes mellitus.
