# Supplementary material for: Loss of microstructural integrity in left hemispheric white matter tracts is associated with poorer digits in noise understanding
Source: GeroScience. 2025 May 26;48(1):1063–77. doi: 10.1007/s11357-025-01707-5 (PMC12972419; doi:10.1007/s11357-025-01707-5)
Supplement: Supplementary file 3 — (DOCX 38.9 KB) [file 11357_2025_1707_MOESM3_ESM.docx]

**Appendix C. Supplementary data**

**Table C.1**

Associations between the SRT and the tract-specific ADs

| WM tracts | | Model I | | | | Model II | | | | | Model III | | | |
| --- | --- | --- | --- | --- | --- | --- | --- | --- | --- | --- | --- | --- | --- | --- |
|  | Left hemisphere | | Right hemisphere | | Left hemisphere | | Right hemisphere | | | Left hemisphere | | | Right hemisphere | |
| *Brainstem tracts* | | | | | | | | |  | | |  | | |
| Middle cerebellar peduncle | | -0.0089  (-0.056, 0.039) | | | | -0.015  (-0.063, 0.033) | | | | | -0.013  (-0.06, 0.034) | | | |
| Medial lemniscus | | -0.027  (-0.076, 0.022) | | -0.027  (-0.076, 0.022) | | -0.025  (-0.075, 0.025) | | -0.025  (-0.074, 0.024) | | | -0.019  (-0.069, 0.031) | | | -0.020  (-0.069, 0.029) |
| *Projection tracts* | | | | | | | | |  | | |  | | |
| Corticospinal tract | | 0.045  (-0.012, 0.10) | | 0.074 *  (0.017, 0.13) | | 0.050  (-0.0082, 0.11) | | 0.074 *  (0.016, 0.13) | | | 0.053  (-0.0051, 0.11) | | | 0.075 *  (0.017, 0.13) |
| Anterior thalamic radiation | | 0.037  (-0.035, 0.11) | | 0.021  (-0.049, 0.091) | | 0.026  (-0.046, 0.098) | | 0.018  (-0.052, 0.088) | | | 0.026  (-0.046, 0.098) | | | 0.016  (-0.054, 0.086) |
| Superior thalamic radiation | | 0.09 **  (0.029, 0.15) | | 0.084 **  (0.024, 0.14) | | 0.087 **  (0.026, 0.15) | | 0.083 **  (0.022, 0.14) | | | 0.086 **  (0.025, 0.15) | | | 0.08 **  (0.019, 0.14) |
| Posterior thalamic radiation | | 0.053  (-0.0029, 0.11) | | 0.024  (-0.032, 0.08) | | 0.059 *  (0.0031, 0.11) | | 0.025  (-0.031, 0.081) | | | 0.061 *  (0.0054, 0.12) | | | 0.020  (-0.035, 0.075) |
| *Association tracts* | | | | | | | | |  | | |  | | |
| Superior longitudinal fasciculus | | 0.035  (-0.022, 0.092) | | 0.024  (-0.035, 0.083) | | 0.032  (-0.026, 0.09) | | 0.026  (-0.033, 0.085) | | | 0.034  (-0.023, 0.091) | | | 0.027  (-0.032, 0.086) |
| Inferior longitudinal fasciculus | | 0.015  (-0.040, 0.070) | | 0.048  (-0.0083, 0.10) | | 0.017  (-0.039, 0.073) | | 0.051  (-0.0055, 0.11) | | | 0.017  (-0.039, 0.073) | | | 0.048  (-0.0082, 0.10) |
| Inferior fronto-occipital fasciculus | | 0.075 **  (0.019, 0.13) | | 0.0021  (-0.053, 0.058) | | 0.081 **  (0.025, 0.14) | | 0.0019  (-0.054, 0.058) | | | 0.083 **  (0.027, 0.14) | | | 0.0035  (-0.052, 0.059) |
| Uncinate fasciculus | | 0.060 *  (0.0057, 0.11) | | 0.065 *  (0.012, 0.12) | | 0.046  (-0.0088, 0.10) | | 0.059 *  (0.006, 0.11) | | | 0.048  (-0.0065, 0.10) | | | 0.061 *  (0.0083, 0.11) |
| *Limbic system tracts* | | | | | | | | |  | | |  | | |
| Cingulate gyrus part of the cingulum | | 0.027  (-0.022, 0.076) | | -0.020  (-0.069, 0.029) | | 0.026  (-0.023, 0.075) | | -0.020  (-0.069, 0.029) | | | 0.026  (-0.023, 0.075) | | | -0.018  (-0.067, 0.031) |
| Parahippocampal part of the cingulum | | 0.042  (-0.0056, 0.09) | | 0.018  (-0.031, 0.067) | | 0.048  (-0.00015, 0.096) | | 0.020  (-0.029, 0.069) | | | 0.047  (-0.00092, 0.095) | | | 0.019  (-0.030, 0.068) |
| *Callosal tracts* | | | | | | | | |  | | |  | | |
| Forceps major | | 0.025 (-0.027, 0.077) | | | | 0.025 (-0.027, 0.077) | | | | | 0.023 (-0.029, 0.075) | | | |
| Forceps minor | | 0.021 (-0.029, 0.071) | | | | 0.023 (-0.027, 0.073) | | | | | 0.025 (-0.025, 0.075) | | | |

Values represent the mean differences in z-score (95% confidence interval) of the SII per standard deviation increase of the tract-specific AD. Stars indicate the significance level: * (p < 0.05), ** (p < 0.01), *** (p < 0.001). Results in bold were statistically significant after correction for multiple testing (p < 0.0032). Model I: adjusted for sex, age, age^2^, PTA, tract-specific WM volume, natural-log-transformed tract-specific WMH volume, ICV, and time between the hearing assessment and brain MRI acquisition. Model II: Model I and additionally adjusted for educational level, smoking behaviour, alcohol consumption, systolic blood pressure, diastolic blood pressure, the use of anti-hypertensive drugs and the presence of diabetes mellitus.

**Table C.2**

Associations between the SRT and the tract-specific RDs

| WM tracts | | Model I | | | | Model II | | | | | Model III | | | |
| --- | --- | --- | --- | --- | --- | --- | --- | --- | --- | --- | --- | --- | --- | --- |
|  | Left hemisphere | | Right hemisphere | | Left hemisphere | | Right hemisphere | | | Left hemisphere | | | Right hemisphere | |
| *Brainstem tracts* | | | | | | | | |  | | |  | | |
| Middle cerebellar peduncle | | 0.0024  (-0.044, 0.049) | | | | 0.0013  (-0.046, 0.048) | | | | | -0.0031  (-0.050, 0.044) | | | |
| Medial lemniscus | | -0.033  (-0.082, 0.016) | | -0.0097  (-0.059, 0.040) | | -0.048  (-0.097, 0.001) | | -0.026 (-0.076, 0.024) | | | -0.044 (-0.093, 0.0048) | | | -0.022 (-0.072, 0.028) |
| *Projection tracts* | | | | | | | | |  | | |  | | |
| Corticospinal tract | | -0.022  (-0.078, 0.034) | | 0.042  (-0.012, 0.096) | | -0.036  (-0.093, 0.021) | | 0.029  (-0.026, 0.084) | | | -0.031  (-0.087, 0.025) | | | 0.029  (-0.026, 0.084) |
| Anterior thalamic radiation | | 0.041  (-0.035, 0.12) | | 0.039  (-0.037, 0.11) | | 0.031  (-0.046, 0.11) | | 0.036  (-0.040, 0.11) | | | 0.030  (-0.047, 0.11) | | | 0.031  (-0.045, 0.11) |
| Superior thalamic radiation | | 0.033  (-0.028, 0.094) | | 0.050  (-0.009, 0.11) | | 0.030  (-0.033, 0.093) | | 0.044  (-0.016, 0.10) | | | 0.034  (-0.028, 0.096) | | | 0.041  (-0.018, 0.10) |
| Posterior thalamic radiation | | **0.086 ****  **(0.03, 0.14)** | | 0.039  (-0.02, 0.098) | | **0.086 ****  **(0.03, 0.14)** | | 0.036  (-0.023, 0.095) | | | **0.084 ****  **(0.028, 0.14)** | | | 0.034  (-0.024, 0.092) |
| *Association tracts* | | | | | | | | |  | | |  | | |
| Superior longitudinal fasciculus | | 0.057  (-0.0017, 0.12) | | 0.014  (-0.045, 0.073) | | 0.054  (-0.0056, 0.11) | | 0.0099  (-0.050, 0.070) | | | 0.055  (-0.0043, 0.11) | | | 0.013  (-0.046, 0.072) |
| Inferior longitudinal fasciculus | | 0.083 **  (0.025, 0.14) | | 0.062 *  (0.0037, 0.12) | | 0.075 *  (0.016, 0.13) | | 0.058  (-0.00079, 0.12) | | | 0.073 *  (0.014, 0.13) | | | 0.055  (-0.0035, 0.11) |
| Inferior fronto-occipital fasciculus | | **0.12 *****  **(0.06, 0.18)** | | **0.036 *****  **(-0.025, 0.097)** | | 0.12  (0.060, 0.18) | | 0.037  (-0.025, 0.099) | | | **0.12 *****  **(0.06, 0.18)** | | | 0.039  (-0.022, 0.10) |
| Uncinate fasciculus | | 0.069 *  (0.013, 0.13) | | 0.050  (-0.0044, 0.10) | | 0.065 *  (0.0078, 0.12) | | 0.048  (-0.0069, 0.1) | | | 0.062 *  (0.0051, 0.12) | | | 0.050  (-0.0046, 0.10) |
| *Limbic system tracts* | | | | | | | | |  | | |  | | |
| Cingulate gyrus part of the cingulum | | -0.0042 (-0.055, 0.047) | | 0.056 *  (0.0065, 0.11) | | -0.0019  (-0.054, 0.05) | | 0.053 *  (0.003, 0.1) | | | 0.0036  (-0.048, 0.055) | | | 0.058 *  (0.0083, 0.11) |
| Parahippocampal part of the cingulum | | 0.03 (-0.017, 0.077) | | 0.012 (-0.036, 0.06) | | 0.028  (-0.019, 0.075) | | 0.015  (-0.033, 0.063) | | | 0.027  (-0.020, 0.074) | | | 0.015  (-0.033, 0.063) |
| *Callosal tracts* | | | | | | | | |  | | |  | | |
| Forceps major | | 0.052 (-0.0067, 0.11) | | | | 0.053 (-0.006, 0.11) | | | | | 0.054 (-0.0047, 0.11) | | | |
| Forceps minor | | 0.043 (-0.014, 0.1) | | | | 0.039 (-0.019, 0.097) | | | | | 0.039 (-0.018, 0.096) | | | |

Values represent the mean differences in z-score (95% confidence interval) of the SII per standard deviation increase of the tract-specific RD. Stars indicate the significance level: * (p < 0.05), ** (p < 0.01), *** (p < 0.001). Results in bold were statistically significant after correction for multiple testing (p < 0.0032). Model I: adjusted for sex, age, age^2^, PTA, tract-specific WM volume, natural-log-transformed tract-specific WMH volume, ICV, and time between the hearing assessment and brain MRI acquisition. Model II: Model I and additionally adjusted for educational level, smoking behaviour, alcohol consumption, systolic blood pressure, diastolic blood pressure, the use of anti-hypertensive drugs and the presence of diabetes mellitus.
